# CONNECTING RURAL OLDER ADULTS WITH DISABILITIES TO HOME MODIFICATIONS WITH A REMOTE HOME ASSESSMENT

**DOI:** 10.1093/geroni/igac059.1991

**Published:** 2022-12-20

**Authors:** Emily Nabors, Catia Garell, Jon Pynoos

**Affiliations:** University of Southern California, Los Angeles, California, United States; Thrive for Life LLC, Honolulu, Hawaii, United States; University of Southern California, Los Angeles, California, United States

## Abstract

Older adults living in rural areas have particular challenges to accessing critical supportive services such as home modifications to promote functioning and safety. Conducting remote home assessments through telehealth has the potential to reduce time spent and overall cost that occur in conducting in-person assessments. During the pandemic, providers turned to telehealth to preserve continuity of assessment services with few research-based practices to guide them. With support from a NIDLRR SBIR grant, Thrive for Life LLC in partnership with the USC Leonard Davis School of Gerontology conducted research to develop a remote home assessment that aims to connect health and home modification providers with rural older adults (65+) with disabilities, a population that may not receive home modifications otherwise. Research included a literature review, key informant interviews with five experts in the field, and individual phone interviews with 30 rural older adults who have disabilities. The literature was analyzed and used to inform the interview questions. Key informant interview responses were analyzed for models, potential challenges, lessons learned, and opportunities to impact priority needs. Consumer interview responses were analyzed for needs, preferences, concerns, and challenges related to technology use. Findings demonstrate common barriers such as lack of access to broadband and smart technology; circumstances in which remote assessments are, and are not, likely to be successful; and the potential value of conducting remote home assessments in rural areas to ensure equity of access to home modifications for older adults with disabilities during the pandemic and beyond.

